# Aggretin Venom Polypeptide as a Novel Anti-angiogenesis Agent by Targeting Integrin alpha2beta1

**DOI:** 10.1038/srep43612

**Published:** 2017-03-02

**Authors:** Ching Hu Chung, Chien Hsin Chang, Chun Chieh Hsu, Kung Tin Lin, Hui Chin Peng, Tur Fu Huang

**Affiliations:** 1 Department of Medicine, Mackay Medical College, New Taipei City, Taiwan; 2 Institute of Pharmacology, College of Medicine, National Taiwan University, Taipei, Taiwan; 3 Medical and Pharmaceutical Industry Technology and Development Center, New Taipei City, Taiwan

## Abstract

VEGF and VEGFR antibodies have been used as a therapeutic strategy to inhibit angiogenesis in many diseases; however, frequent and repeated administration of these antibodies to patients induces immunogenicity. In previous studies, we demonstrated that aggretin, a heterodimeric snake venom C-type lectin, exhibits pro-angiogenic activities via integrin α2β1 ligation. We hypothesised that small-mass aggretin fragments may bind integrin α2β1 and act as antagonists of angiogenesis. In this study, the anti-angiogenic efficacy of a synthesised aggretin α-chain C-terminus (AACT, residue 106–136) was evaluated in both *in vitro* and *in vivo* angiogenesis models. The AACT demonstrated inhibitory effects on collagen-induced platelet aggregation and HUVEC adhesion to immobilised collagen. These results indicated that AACT may block integrin α2β1−collagen interaction. AACT also inhibited HUVEC migration and tube formation. Aortic ring sprouting and Matrigel implant models demonstrated that AACT markedly inhibited VEGF-induced neovascularisation. In addition, induction of FAK/PI3K/ERK1/2 tyrosine phosphorylation and talin 1/2 associated with integrin β1 which are induced by VEGF were blocked by AACT. Similarly, tyrosine phosphorylation of VEFGR2 and ERK1/2 induced by VEGF was diminished in integrin α2-silenced endothelial cells. Our results demonstrate that AACT is a potential therapeutic candidate for angiogenesis related-diseases via integrin α2β1 blockade.

Angiogenesis is the growth of blood vessels from pre-existing vasculature and plays an important role in wound healing, tumour growth/metastasis and inflammation-related diseases^1^. Accordingly, there has been considerable interest in the use of novel anti-angiogenic agents as adjuncts to cancer therapies^2^. Endothelial cells interact with the extracellular matrix (ECM) through cell surface adhesion receptors that mediate the neovascularisation processes^3^. β1 and αv integrins have been reported to modulate neovascularisation processes, and αvβ3 has also been implicated in angiogenesis due to its high level of expression in angiogenic vessels^4^. The role of these adhesion molecules in angiogenesis is demonstrated by the *in vivo* anti-angiogenic efficacy of αvβ3 monoclonal antibodies and αvβ3 antagonists including the snake venom disintegrin, which has demonstrated anti-angiogenic efficacy *in vivo*^5^.

Collagen is one of the ECM and is crucial for cell migration^6^. Integrin α2β1, one of several collagen receptors, is expressed on endothelial cells and platelets. Upon integrin α2β1-expressing cell adhesion to collagen, many physiological functions are activated, including extracellular matrix remodelling and the ERK pathway^7^. α2β1 integrin has been implicated in extracellular matrix remodelling in addition to endothelial cell migration, proliferation and neovascular formation^8^. Snake venoms contain many enzymes and polypeptides which can affect the matrix and cell interaction^9^. We previously demonstrated that a C-type lectin-related protein, aggretin, exhibits pro-angiogenic activities through interaction with endothelial integrin α2β1 as a collagen-like agonist^10^. Using binding and functional studies, we demonstrated that integrin α2β1 is the major receptor of aggretin on human umbilical vascular endothelial cell (HUVECs)^11^. *In vivo* vascular endothelial growth factor (VEGF)-driven angiogenesis was selectively reduced by integrins α1 and α2 inhibition without affecting any pre-existing vasculature^12^. In addition, one selective α1β1 integrin inhibitor, obtustatin, has been reported to inhibit *in vivo* angiogenesis^13^. These data indicate that integrin α2β1 and α1β1 antagonism may inhibit signalling pathways involved in angiogenesis.

VEGF has been established to be involved in many stages of angiogenesis in malignant diseases via its multi-functional effects in activating and integrating signalling pathway networks^14^. VEGF signalling blockade reduces new vessel growth and induces endothelial cell apoptosis. Thus, the use of tyrosine kinase inhibitors or VEGF/VEGF receptor (VEGFR) antibodies to inhibit crucial angiogenic steps represents a practical therapeutic strategy for the treatment of neovascularisation diseases^15^. E7820, a potent angiogenesis inhibitor, has been shown to reduce integrin α2 mRNA expression and inhibit basic fibroblast growth factor/VEGF-induced HUVEC proliferation and tube formation^16^,^17^. Integrin α2β1/α1β1 expression is reportedly regulated by VEGF, and an inhibitory antibody against α2β1/α1β1 has been shown to inhibit angiogenesis and tumour growth in VEGF-overexpressing tumour cells^12^,^18^. Therefore, we hypothesised that peptide-based integrin α2β1 blockade may have potential anti-tumour effects by inhibiting angiogenesis.

In this study, we demonstrate that aggretin α-chain C-terminal (AACT, 31 amino acid residues) inhibits collagen-induced platelet aggregation and HUVEC adhesion predominantly via integrin α2β1 ligation. The ability of endothelial cells to adhere to collagen was also diminished by integrin α2 silencing. Thus, we hypothesised that aggretin-derived integrin α2 antagonism may inhibit angiogenesis in response to VEGF. In this study, we unveiled the anti-angiogenic activities of AACT by demonstrating its inhibitory effects on HUVEC migration, Matrigel-induced capillary tube formation and aortic ring sprouting in *ex vivo* assays and reducing neovascularisation in Matrigel implant angiogenesis assays *in vivo*. VEGF-stimulated focal adhesion kinase (FAK), Phosphoinositide 3-kinase (PI3K) and Extracellular Signal-regulated Kinase 1/2 (ERK 1/2) phosphorylation were attenuated by AACT. The talin1/2 associated with integrin β1 was also abolished by AACT. Similarly, VEGF-induced VEFGR2 and ERK1/2 activation were abolished by integrin α2 siRNA transfection. These results demonstrate that AACT inhibits angiogenesis in response to VEGF via α2β1 integrin blockade.

## Results

### Effects of AACT on collagen-induced platelet aggregation and HUVEC-collagen interaction

**Figure 1:**
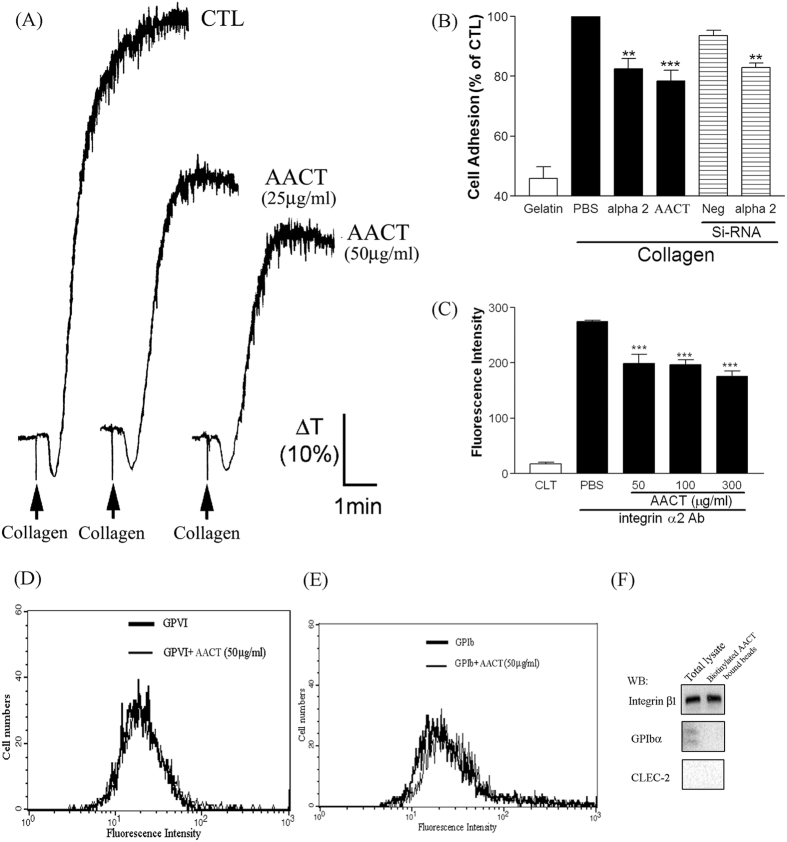
Effects of AACT on collagen-induced platelet aggregation and HUVEC-collagen interation. (**A**) Washed platelets were preincubated with AACT (25, 50 μg/ml) at 37 °C for 3 min, and then collagen (3 μg/ml) was added to trigger platelet aggregation. Platelet aggregation was measured turbidimetrically (ΔT, change in transmission). All experiments were conducted in triplicate at least four times and similar results were obtained. (**B**) HUVECs were seeded onto plates coated with collagen or negative control (gelatin). Cells were labeled with fluorescent dye BCECF-AM for 30 min and then preincubated with integrin α2 Ab, AACT or pre-transfected with integrin α2 siRNA. Attached cells were read by Cytofluor microplate reader with fluorescence excitation and emission wavelength at 485 nm and 530 nm, respectively, and were quantified as the percentage of fluorescence intensity of control. HUVECs were preincubated with vehicle or AACT (50, 100, 300 μg/ml in C; 50 μg/ml in D and E) for 30 min, then the binding as probed with anti-integrin α2 (**C**), anti-GPVI (**D**) or anti-GPIb (**E**) mAb and subjected to flow cytometric analysis by using FITC-conjugated anti-IgG mAbs as the second antibody. (**F**) HUVEC proteins eluted from biotinylated AACT-bound streptavidin–Sepharose beads were blotted with anti-integrin β1, GPIbα and CLEC-2 antibodies. Results are presented as cell numbers vs. binding fluorescence intensity. Data are presented as mean ± S.E.M. (n = 4). **P < 0.01, ***P < 0.001 compared with control. The pattern shown is a representative of one of at least three similar results.

Since the integrin β1/C-type lectin-like receptor 2 (CLEC-2) were demonstrated as the binding targets of AACT^19^ and there are lack of CLEC-2 expression in HUVECs^20^, the integrin α2β1 may be the binding target in HUVECS. To investigate the inhibitory effect of AACT on integrin α2β1 activation, we examined the effect of AACT on collagen-induced platelet aggregation. As shown in Fig. 1A, AACT (25 and 50 μg/ml, equivalent to 6.75 and 13.5 μM, respectively) significantly inhibited collagen-induced aggregation (approximately 50% inhibition). Furthermore, to confirm integrin α2β1 as the major target for AACT-mediated HUVEC-collagen attachment, we next examined the involvement of integrin α2 in cell adhesion. Endothelial cell adhesion to collagen was inhibited by integrin α2 mAb and AACT (50 μg/ml). Similarly, knockdown of α2 also inhibited cell adhesion to collagen (Fig. 1B). Moreover, we investigated the binding of AACT to integrin α2. HUVECs treated with or without AACT (50, 100 and 300 μg/ml) were cultured with anti- α2 antibodies. As shown in Fig. 1C, AACT inhibited the binding of integrin α2 mAb to endothelial cells as measured by flow cytometry, but not the binding of anti- Glycoprotein VI (GPVI) or anti-Glycoprotein Ib (GPIb)(AP1) antibodies (Fig. 1D and E). We also used the HUVECs membrane receptor to explore the binding site of AACT on HUVECs. HUVECs membrane proteins bound to biotinylated AACT were isolated and eluted. Only one membrane receptor was recognized by integrin β1 (Fig. 1F). These results indicate that AACT inhibits platelet and HUVEC-collagen adherence, predominantly via integrin α2 blockade.

### Effects of AACT on HUVEC viability and proliferation

**Figure 2:**
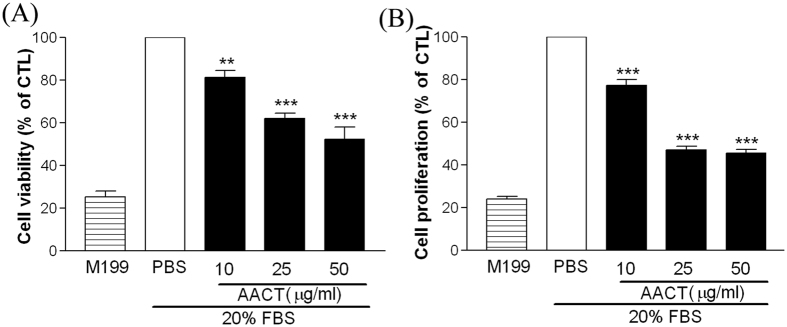
Effects of AACT on HUVEC viability and proliferation. HUVECs were seeded overnight for attachment. After a further 16 h of starvation, cells were incubated with medium in the absence (M199) or presence of 20% FBS. Cells were either treated with 20% FBS only as a control or with the indicated concentration of AACT (10, 25, 50 μg/ml) for assay. With 48 h treatment, cells were added (**A**) MTT or (**B**) BrdU reagent. All experiments were conducted in triplicate at least four times and similar results were obtained. Data are presented as mean ± S.E.M. (n = 4). **P < 0.01; ***P < 0.001 compared with the control.

As integrin α2β1 activation is involved in endothelial cell growth, we evaluated the inhibitory effects of AACT on cell viability using 3-[4, 5-dimethylthiazol-2-yl]-2, 5-diphenyltetrazolium bromide (MTT) assays. As shown in Fig. 2A, AACT reduced serum induced HUVEC viability by 63.8% at a concentration of 50 μg/ml. Furthermore, in order to confirm the inhibitory effects of AACT on endothelial cell growth, we performed bromodeoxyuridine assays. As expected, AACT was found to significantly inhibit HUVEC proliferation (Fig. 2B).

### AACT inhibits migration of HUVECs *in vitro* and *ex vivo*

**Figure 3:**
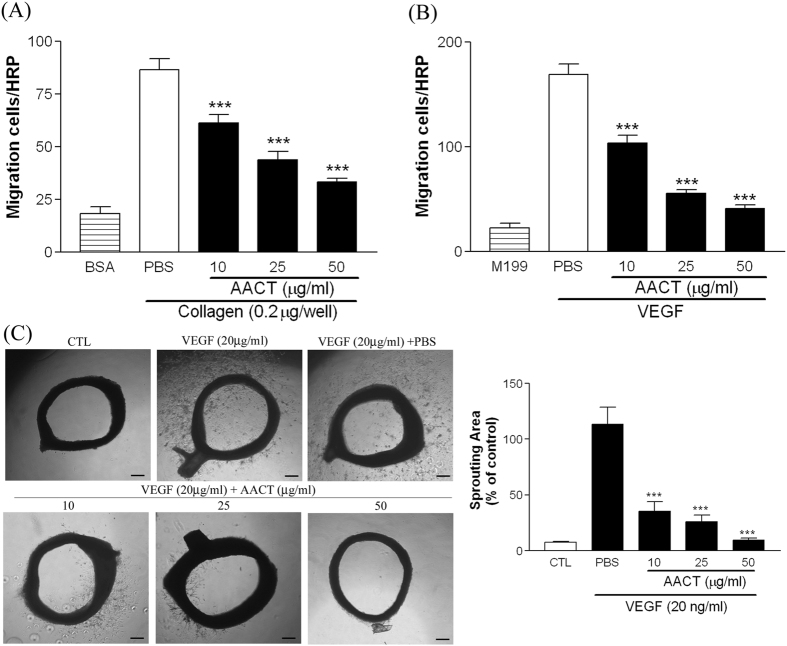
AACT inhibits migration of endothelial cells *in vitro* and *ex vivo*. (**A**) HUVECs (5 × 10^4^/ml) were treated with or without AACT (10, 25, 50 μg/ml) and placed in the upper chamber of a Transwell containing a collagen–coated filter membrane for 16 h. (**B**) HUVECs (5 × 10^4^/ml) were treated with or without indicated concentrations of AACT (10, 25, 50 μg/ml) and placed in the upper chamber of a Transwell containing a gelatin–coated filter membrane. Chemotaxis was induced by VEGF (20 ng/ml) in the lower chamber for 16 h. After removal of non-migrated cells and fixation, cells that migrated to the underside of the filter membrane were stained and quantified by phase-contrast light microscope under a high-power field (HPF; magnification, 100x). (**C**) Aortas in Matrigel were treated with or without AACT (10, 25 or 50 μg/ml) in the VEGF (20 ng/ml) medium. After 8 days, aortic rings were photographed (Scar bar = 100 μm). Experiments were repeated three times and a representative result is shown. All experiments were conducted in triplicate and similar results were repeated four times. Data are presented as mean ± S.E.M. (n = 4). ***P < 0.001 compared with the control.

As HUVECs migration is essential for angiogenesis, the effect of AACT (10, 25 and 50 μg/ml) on HUVEC haptotaxis migration with Transwell was assayed. As shown in Fig. 3A, a 4.72-fold increase was observed in the number of HUVECs in the lower filter membrane coated with collagen. Under similar conditions, AACT significantly inhibited HUVEC migration. Furthermore, we evaluated chemotactic migration with Transwell to determine the effect of AACT on HUVEC migration in response to VEGF. As shown in Fig. 3B, a 7.45-fold increase in the number of HUVECs was observed following VEGF stimulation, with AACT found to inhibit HUVEC migration. In addition, the vessels sprouting of the rat aortic ring induced by VEGF was also significantly decreased in AACT treated group (Fig. 3C). These results showed that AACT is capable of inhibiting VEGF-induced HUVECs migration *in vitro* and *ex vivo*.

### Effects of AACT on Matrigel tube formation

**Figure 4:**
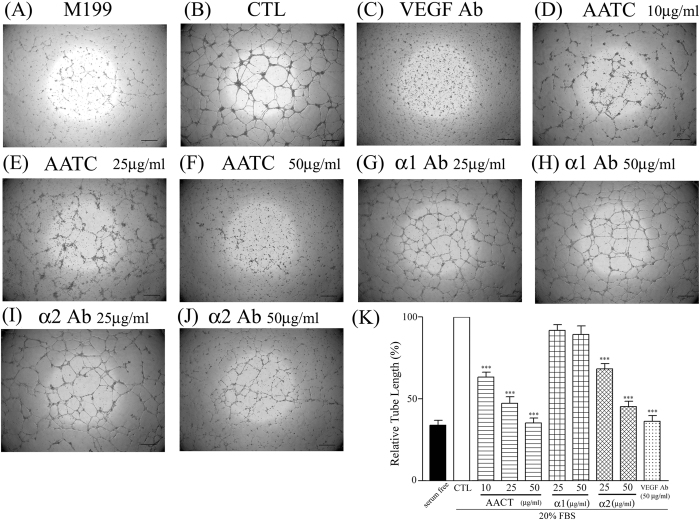
Effects of AACT on Matrigel tube formation. HUVECs (1.2 × 10^5^/well) were placed on Matrigel for 16 h in the absence (**A**) or presence of 20% FBS (**B**) In inhibitory studies, HUVECs were pretreated with VEGF Ab (**C**), AACT (10, 25, 50 μg/ml, D-F), integrin α1 Ab (25, 50 μg/ml, G and H), integrin α2 Ab (25, 50 μg/ml, I and J). After washing and fixation, cells were observed under the microscope at 40x magnification and photographed (Scar bar = 100 μm). Quantitative analyses for tube length were presented as fold-change relative to presence of 20% FBS control (**K**) The pattern shown is a representative of one of at least three similar results. Data are presented as mean ± S.E.M. (n = 4). ***P < 0.001 compared with the control.

HUVECs had significantly greater numbers of branching tube networks after 16 h of 20% FBS incubation (20% FBS treatment Fig. 4B as compared to serum free Fig. 4A), and this tube branching was attenuated by VEGF Ab treatment (Fig. 4C). AACT (10, 25 and 50 μg/ml) also attenuated serum-induced HUVEC tube formation (Fig. 4D–F). Moreover, to confirm the involvement of integrin α2 in the tube formation process, we examined the inhibitory effect of integrin α1 and α2 Ab in our *in vitro* angiogenic model. Integrin α2 mAb treatment, but not integrin α1 mAb treatment, significantly decreased VEGF-induced tube formation (Fig. 4G–J). These results indicate that AACT inhibits VEGF-stimulated angiogenesis predominantly via integrin α2 blockade, as shown in Fig. 4K.

### Effect of AACT on angiogenesis in response to Matrigel implantation

**Figure 5:**
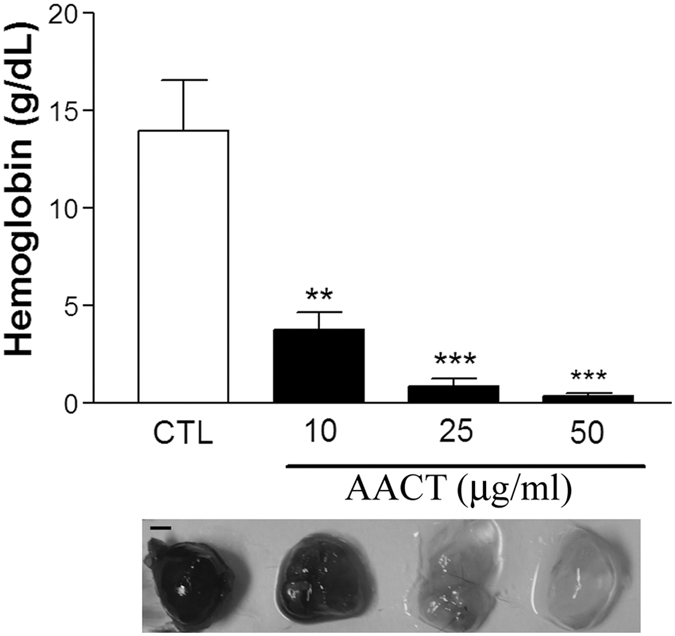
AACT inhibits VEGF-induced angiogenesis in mouse Matrigel-plug assay. Matrigel 500 μl containing 200 ng/ml VEGF with or without AACT (10, 25, 50 μg/ml) was subcutaneously injected into C57BL/5 C mice (5–9 mice/group). After 7 days, plugs were taken and photographed (Scar bar = 2 mm). Hemoglobin was measured as an indication of blood vessel formation, using the Drabkin method. Data are presented as mean ± S.E.M of at least 3 mice per group. **P < 0.01, ***P < 0.001 compared with control.

An *in vivo* model containing Matrigel premix with VEGF (200 ng/ml) was used to determine the inhibitory effect of AACT on angiogenesis. Matrigel (in the presence or absence of AACT (10, 25 and 50 μg/ml)) was then subcutaneously injected into mice. At 7 days after inoculation, capillary network formation was observed in implanted plugs. In the AACT-treated group, less vessel growth and less red blood cell infiltration was observed in implanted plugs (Fig. 5). Haemoglobin levels were significantly lower in AACT-treated mice. These results suggest that AACT also inhibits angiogenesis *in vivo*.

### Effect of AACT on FAK/PI3K/ERK1/2 activation and talin 1/2 associated with integrin β1

**Figure 6:**
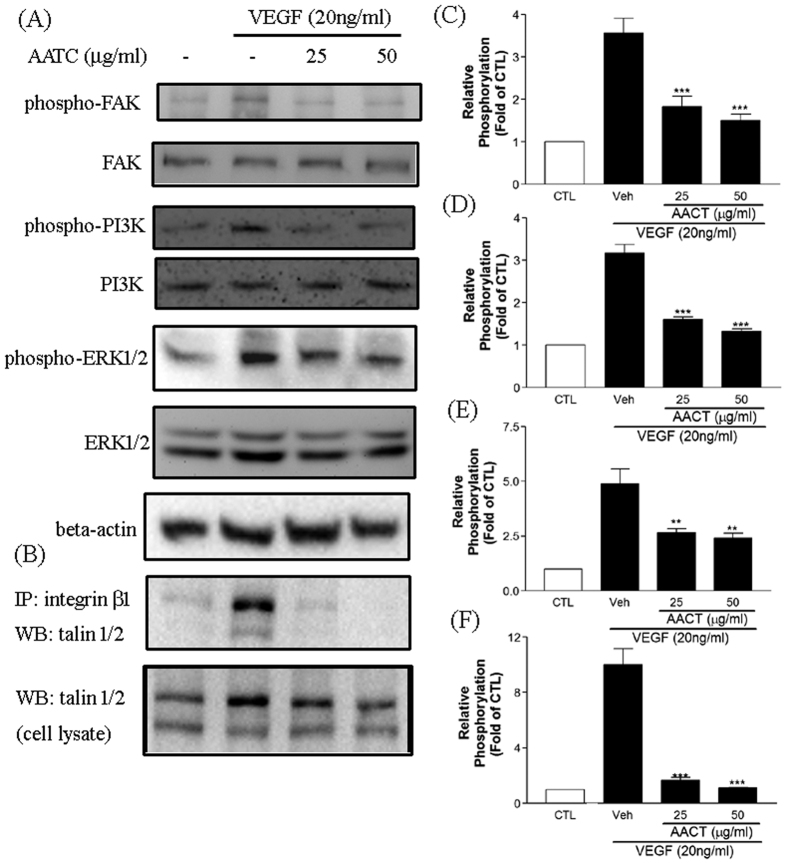
Effect of AACT on FAK, ERK1/2 and PI3K p85α activation and talin1/2 associated with integrin β1. (**A**) HUVECs were pretreated with or without indicated concentrations of AACT for 30 min and vehicle or VEGF (20 ng/ml) was added to the cells as a basal or control for another 10 min. Cells were lysed with lysis buffer. FAK, PI3K and ERK1/2 activation were detected by Western blotting using anti-phospho-FAK, anti-phospho-PI3K p85α and anti-phospho-ERK1/2 mAb, whereas beta actin was used as an internal control. (**B**) HUVECs were pretreated with or without indicated concentrations of AACT for 30 min and vehicle or VEGF (20 ng/ml) was added to the cells as a basal or control for another 10 min. Cells were lysed with lysis buffer and immunopreciptated with integrin β1 mAb. Talin 1/2 associated with integrin β1 were detected by Western blotting using anti talin 1/2 mAb. Blot images were cropped for comparison and all relevant gels have been run under similar experimental conditions. The pattern is one example of three independent experiments with similar results. Quantitative analyses of FAK (**C**), PI3K p85α (**D**) and ERK1/2 (**E**) tyrosine phosphorylation are presented as mean density for the ratio between phosphorylated protein and total protein as determined by a densitometer. Quantitative analyses of talin 1/2 association are presented as mean density for the ratio between associated protein and total protein as determined by a densitometer (**F**). **I**ntensities were normalized to static basal, and fold decreases were calculated. Data are presented as mean ± S.E.M. (n = 4). ***P < 0.001 compared with control. The pattern shown is representative of 1 of at least 3 similar results.

FAK and PI3K phosphorylation are involved in many cell responses to VEGF. In this study, we found that VEGF-induced FAK and PI3K p85α phosphorylation were significantly inhibited by AACT (Fig. 6). AACT pre-treated HUVECs also demonstrated significantly decreased ERK1/2 activation (Fig. 6). Integrins are well established to be activated by clustering and binding of talin to integrin β-tail, we further tested the talin 1/2 association with integrin β1. Talin 1/2 and integrin β1 association was significantly abolished by AACT treatment. These results suggest that AACT inhibits integrin a2β1 activation and reduces VEGF–stimulated FAK, PI3K and ERK signalling.

### Role of integrin α2 involved in VEGF signalling

**Figure 7:**
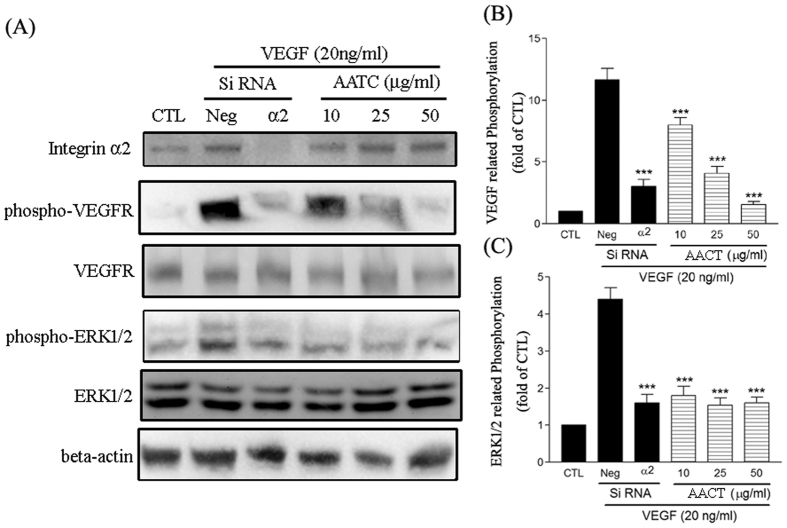
Involvment of integrin α2 in VEGF signalling. (**A**) Endothelial cells were treated with or without VEGF (20 ng/ml) for 10 min. Cells were lysed with lysis buffer. VEGFR2 and ERK1/2 activation was detected by Western blotting using anti-phospho-VEGFR2 mAb or anti-phospho-ERK mAb, whereas beta actin was used as an internal control. Integrin α2 expression after siRNA interference was detected by Western blotting using anti-integrin α2 mAb. Blot images were cropped for comparison and all relevant gels have been run under similar experimental conditions. The pattern is one example of three independent experiments with similar results. Quantitative analyses of VEGFR2 (**B**) and ERK1/2 (**C**) tyrosine phosphorylation are presented as mean density for the ratio between phosphorylated protein and total protein as determined by a densitometer. Intensities were normalized to static basal, and fold decreases were calculated. Data are presented as mean ± S.E.M. (n = 4). ***P < 0.001 compared with the control.

The integrin α2-subunit siRNA was used to evaluate the involvement of integrin α2β1 in VEGF signalling. Expression levels of integrin α2 markedly decreased in siRNA-transfected cells compared to non-targeted control siRNA-transfected HUVECs (Fig. 7). VEGF-induced VEGF Receptor 2 and its downstream signalling molecular ERK1/2 phosphorylation were significantly inhibited in both integrin α2 siRNA transfected endothelial cells and cells pre-treated with AACT (Fig. 7). Collectively, these results indicate that integrin α2 is involved in VEGF signalling and that AACT inhibits angiogenesis via endothelial integrin α2 ligation.

## Discussion

HUVEC expression of integrin αVβ3, αVβ5 and α2β1 has been implicated in angiogenesis^21^,^22^. Several snake venoms interact with platelets via α2β1, GPVI and GPIb; however, few demonstrate significant specificity. Many snake venoms have binding sites for several platelet targets. Recombinant techniques allow the production of specific polypeptides which bind integrin α2β1 without involving GPIb or GPVI. In previous studies, we demonstrated that aggretin induces platelet activation through binding integrin α2β1 and GPIb leading to FAK and PLCγ2 phosphorylation^23^. Aggretin also induces HUVEC-dependent angiogenesis by interacting with integrin α2β1 through the PI3K/Akt/ERK1/2 pathways, with increased expression of VEGF^11^. Therefore, in this study we synthesised a small-mass aggretin fragment, AACT, and examined its effects on platelet aggregation and angiogenesis. Interestingly, we demonstrated that AACT blocks platelet aggregation induced by collagen (Fig. 1A), HUVEC-collagen attachment and HUVEC-integrin α2 mAb binding (Fig. 1B,C). These results indicate that this aggretin fragment acts as α2β1 antagonist rather than an intrinsic α2β1 agonist such as intact aggretin. Similarly, AACT was found to inhibit collagen/VEGF-induced HUVEC migration, FBS-induced Matrigel tube formation and VEGF-induced aortic ring sprouting (Figs 2, 3, 4). Since integrin α2β1 but not GPIb, GPVI and CLEC-2 is the major target of aggretin on endothelial cells (Fig. 1D–F), we hypothesised that AACT blocks *in vitro* or *ex vivo* angiogenesis via integrin α2β1 ligation. Furthermore, AACT abolished VEGF-induced angiogenesis in a Matrigel plug implant assay, suggesting that AACT may be utilised as an anti-angiogenic peptide for inhibiting angiogenesis *in vivo* (Fig. 5). Although the AACT exhibited a potent antiangiogenic activity, most results in our study is not dose-dependent. According to the previous studies, there are several sites for collagen/snacle binding and induced α2β1 activation^24^,^25^,^26^. It may be the reason why AACT was failed to show dose-dependent effect via competitive inhibition. We also found other native peptides derived from integrin α2β1 inhibitor were more likely through the on/off pattern to inhibit integrin α2β1 activity^27^,^28^. The detail integrin α2β1 binding site for AACT still needs further investigation.

Tumour-induced angiogenesis is critical for nutrition and oxygen supply via blood to local tumour and tumour metastases to other organs. Anti-angiogenesis therapy provides many benefits, including broad applicability to different tumour types, less tumour cell resistance and reduced chemotherapeutic dosages. A C-type lectin-like selective α2β1 integrin inhibitor, vixapatin, has demonstrated the ability to inhibit angiogenesis^29^. E7820 inhibits the proliferation and tube formation of HUVECs through suppression of endothelial integrin α2 mRNA expression^30^. These studies indicate that integrin α2β1 mediated angiogenesis may represent a novel pharmacological target.

VEGF is the major angiogenesis regulatory factor and induces neovascularisation via interaction with endothelial cells^31^. To regulate angiogenesis-related processes, VEGF activates many signal transduction networks, such as FAK, PI3K/AKT, ERK1/2, Src and PLCγ. The inhibitory effect of AACT may be through ligation to integrin α2β1, resulting in blockade of the VEGF signal transduction pathway. VEGF-induced PI3K and ERK1/2 activation were markedly inhibited by pre-treatment of HUVECs with AACT in this study. Several β1 integrin have been found to regulate angiogenesis and VEGFR2 activity^32^,^33^. β1 integrins and VEGFR2 interaction plays a role in infantile hemangiomas pathogenesis and matrix-bound VEGFA signalling^34^,^35^. The CD36 and β1 integrin association was reported to cooperate with VEGFR2 in promoting angiogenesis^36^. Integrin α2β1 also physically associates with EGFR and functions regulate EGFR activation^37^. These findings support a role for integrin α2β1 as a mediator for VEGFR2 signalling. To confirm the action of integrin α2β1 in VEGF induced signalling, we transfected integrin α2 siRNA and evaluated the effect of VEGF, where VEGF-induced ERK1/2 activation was found to be significantly inhibited by integrin α2 siRNA (Fig. 7A). These results suggest that the existence of a crosstalk or a positive feedback loop between integrin β1 and VEGFR signaling.

The modulatory role of integrin α2β1 in angiogenesis observed in *in vitro* experiments illustrates its involvement in supporting VEGF signalling and HUVEC migration. Studies from other researchers have provided additional support for the involvement of α2β1^12^,^13^. Although the significance of the integrin α2β1 in tissue angiogenesis remains undetermined, our findings support the concept that integrin α2β1 contributes to the regulation of VEGF signalling. Integrin-linked kinase (ILK) has been identified as an integrin β1 tail binding protein^38^. Silencing ILK with siRNA significantly suppressed tube formation and reduced the tube lengths^39^. Loss of ILK signalling may be involved in decreasing the responses of integrin α2β1-silenced HUVEC to VEGF.

Integrin/ECM interactions are one of the major mediators of cell adhesion-mediated drug resistance^6^. β1 integrins reportedly play a crucial role in head and neck squamous cell carcinoma cell radioresistance^40^. Kanda *et. al.* also reported increased β1, α2 and/or α5 integrin expression in refractory tumours following treatment with gefitinib and/or erlotinib^41^. Furthermore, they demonstrated the integrin β1/Src/Akt signalling pathway as a key mediator of acquired resistance to EGFR-targeted anti-cancer drugs (gefitinib or erlotinib)^41^. Combined treatment with E7820, an integrin α2 expression blocker, and erlotinib significantly decreased microvessel density and increased apoptosis of tumour-associated endothelial cells compared with use of only one of the agents^42^. Ligation of integrin α2β1 may modulate EGFR-mediated endothelial cell functions. The combination of an integrin α2β1 blocker with a growth factor inhibitor may represent an alternative strategy for overcoming drug resistance in cancer treatment.

In summary, we identified an important functional cooperativity between integrin α2β1 and VEGF. In particular, the findings of this study indicate that integrin α2β1 provides crucial support not only for endothelial cell migration but also for VEGF signal transduction and pro-angiogenic functions. In contrast to the intact aggretin, a pro-angiogenic α2β1 agonist, AACT, was found to act as an antagonist of integrin α2β1 and suppresses VEGF-driven angiogenesis in *ex vivo* and *in vivo* models. Many angiogenesis inhibitors have been approved for clinical use and have been studied in clinical trials; however, side effects are reportedly associated with increased risk of arterial thromboembolism^43^. AACT has anti-angiogenic potential and exhibits anti-thrombotic activity by blocking collagen-induced platelet activation. This may also represent an alternative advantage of AACT for patients receiving anti-angiogenesis therapy at a risk of thromboembolism. Recently, our group found that AACT also blocks the interaction of platelet receptor CLEC-2 with the tumour receptor, podoplanin^19^. CLEC-2, a type II transmembrane receptor, is highly and selectively expressed in the liver and some myeloid subsets^44^. As CLEC-2 is not expressed on the surface of HUVEC^20^, the possibility of AACT interacting with CLEC2 can be excluded, indicating that integrin α2β1 is the major target of AACT affecting angiogenesis. Thus, optimisation of small-mass CLP-derived integrin α2β1 antagonists contributes to future drug development.

## Materials and Methods

### Materials

Anti Antiphospho-ERK1/2 (Tyr 204, sc-101761), ERK1/2 (sc-514302), Antiphospho-PI3K-p85α(Tyr 508, sc–12929), PI3K-p85α (sc–1637), p-FAK (Tyr 397, sc-11765-R), FAK (sc-1688), VEGFR-2 (sc-6251) and secondary antibody were purchased from Santa Cruz Biotechnology (Santa Cruz, CA). Anti Antiphospho-VEGFR 2 (Y1175) was purchased from Cell Signaling (Danvers, MA). Beta actin (NB600–501) was purchased from Novvsbio. Iintegrin α2 (ab24697), α1 (ab78479) and β1 (ab30483) were purchased from ABCAM. MTT and toluidine blue O were purchased from Sigma (St Louis, MO). M199, fetal bovine serum (FBS) and other cultured reagents were purchased from Gibco (Grand Island, NY). Endothelial cell growth supplement was purchased from Upstate Biotechnology. Recombinant human VEGF was purchased from R&D Systems (Minneapolis, MN). Aggretin α–chain C-terminus (AACT) 106–136 (CGALEKLTGFRKWVNYYCEQMHAFVCKLLPY) were synthesized by MDBio, Inc., Taiwan. All protocol were approved by *Institutional Review of Board, National Taiwan University Hospital* or *Laboratory Animal Center, College of medicine, National Taiwan University*. All experiments were performed in accordance with *College of medicine, National Taiwan University* regulations and the the written informed consent was obtained from all subjects for human platelet suspension preparation.

### Preparation of human platelet suspension and platelet aggregation assay

Platelet suspensions were prepared as previously described^45^.

### HUVECs culture

HUVECs were provided by the National Research Program for Biopharmaceuticals, Taiwan and approved by its *Institutional Review of Board.* HUVECs were maintained in M199 (with FBS (20%), ECGS (30 μg/mL), L-glutamine (4 mM), penicillin (100 U/mL), and streptomycin (100 μg/mL)) and incubated at 37 °C in 5% CO2. The cells were used between second to fourth passages.

### Adhesion Assay

HUVECs were labeled BCECF-AM and then incubated with or without AACT (50 μg/ml) for 37 °C 30 min. HUVECs were applied to plates which were pre-coated with 100 μl matrices (collagen or gelatin, 50 μg/ml) and were subjected to adhesion as previously described^11^.

### Flow cytometry

The inhibitory effect of AATC on integrin α2, GPIb and GPVI interaction with HUVECs was measured by flow cytometry. HUVECs were pretreated with AACT, and incubated with anti-integrin α2, anti-GPVI or anti-GPIb antibody at room temperature, then were analyzed byFACS Calibur (Becton Dickinson, San Diego, CA, USA).

### MTT assay

HUVECs were starved with M199 contain 2% FBS and then grown in M199 with 20% FBS in absence or presence of AACT (10, 25 or 50 μg/ml). HUVECs were subjected to adhesion as previously described^11^.

### BrdU proliferation assay

HUVECs were starved and then treated with or without AACT (10, 25 or 50 μg/ml) for 48 hours. Cell proliferation was measured by BrdU Cell Proliferation Assay Kit (Chemicon, Temecula, U.S.A), and followed by the manufacturer’s protocol.

### Binding assays

HUVECs were fixed with 1% paraglutaldehyde and then pretreated with AACT. Treated cells then incubated with integrin α2 antibody and were subjected to adhesion as previously described^11^.

### Matrigel capillary tube formation

HUVECs (1.2 × 10^5^ cells) treated with or without AACT (10, 25, 50 μg/ml) in presence of 20% FBS. Cells were subjected to tube formation as previously described^11^. Total length in each condition was quantified by using Kurabo Angiogenesis Image Analyzer (Kurabo, Japan).

### Aortic ring sprouting assay

Sprague-Dawley rats aortic rings were treated with or without AACT (10, 25 or 50 μg/ml) and placed in pre-coated Matrigel. Aortic rings were subjected to sprouting assay as previously described^46^. The sprouting area was measured by the area of endothelial cell out-growth.

### Matrigel-implant angiogenesis assay

Matrigel implant assay was modified from previously study^47^. Matrigel containing VEGF (200 ng/ml) was premix with or without AACT were subjected to matrigel implant assay as previously described^48^.

### Migration assay

Transwell (8.0 μm pore size, Costar) were coated with type I collagen (0.2 μg)/BSA (20 μg) or filled absence or presence of VEGF (20 ng/ml) as a chemoattractor in lower chamber. HUVECs were subjected to migration assay as previously described^49^.

### Protein-based affinity pulldown

These studies were performed as previously described^50^.

### Transfection of small interfering RNA (siRNA)

Integrin α2 siRNA or non-targeting siRNA (negative siRNA) were transfected into HUVECs. The integrin α2 sense sequences of these three siRNA sequences were as follows: UGAAUUGUCUGGCGUAUAATT, CAACUGGGAUCUGUUCUGATT and GCCAAUGAGCCGAGAAUUATT. The sequence for the negative siRNA is UAAGGCUAUGAAGAGAUAC

### Immunoprecipitation and immunoblot analysis

HUVECs were pretreated with or without AACT (25 or 50 μg/ml) for 30 min, and VEGF (20 ng/ml) was added to the cells as basal or control for another 10 min. Cells were subjected to immunoprecipitation and immunoblot as previously described^11^.

### Statistical analysis

Data are presented as mean ± SEM. Groups were assessed by one-way ANOVA and Newman–Keuls multiple comparison test. P values less than 0.05 (P < 0.05) were considered to be significantly different.

## Additional Information

**How to cite this article:** Chung C. H. *et al*. Aggretin Venom Polypeptide as a Novel Anti-angiogenesis Agent by Targeting Integrin alpha2beta1. *Sci. Rep.* **7**, 43612; doi: 10.1038/srep43612 (2017).

**Publisher's note:** Springer Nature remains neutral with regard to jurisdictional claims in published maps and institutional affiliations.
